# High‐definition transcranial direct current stimulation—An open‐label pilot intervention in alleviating depressive symptoms and cognitive deficits in late‐life depression

**DOI:** 10.1111/cns.13253

**Published:** 2019-10-28

**Authors:** Hau‐lam Wong, Wai Chi Chan, Yiu‐lung Wong, Sze‐nga Wong, Hui‐yan Yung, Sau‐man Corine Wong, Pak‐wing Calvin Cheng

**Affiliations:** ^1^ Department of Psychiatry The University of Hong Kong Hong Kong; ^2^ Department of Psychiatry Queen Mary Hospital Hong Kong

**Keywords:** aged, cognitive dysfunction, depression, transcranial direct current stimulation

## Abstract

The efficacy of high‐definition transcranial direct current stimulation (HD‐tDCS) in late‐life depression (LLD) remains unknown due to limited research on its therapeutic effects on the hallmarks of LLD—the depressive and cognitive symptoms. The present open‐label pilot study aimed to examine the effectiveness of HD‐tDCS as an augmentation therapy with antidepressants in improving the depressive and cognitive symptoms for LLD. Significant improvements were hypothesized in the depressive, cognitive, and daily functioning outcomes over time. A total of 15 subjects with LLD (13 females, mean age = 73.27 ± 6.25) received five consecutive daily sessions of 20‐minute active HD‐tDCS interventions weekly for 2 weeks, with a 2 mA anodal stimulation over F3 and cathodal stimulation over FC1, AF3, F7, and FC5. Depressive symptoms and cognitive and daily functioning were assessed across five assessment timepoints. The results revealed that the HD‐tDCS was effective in reducing the depressive severity and the remission rates, with a sustained effect at both the 1‐month and 3‐month follow‐up. Pre‐post improvements were seen in the overall cognitive functioning and in verbal fluency, but not in executive functioning. Our pilot study provides a preliminary result of HD‐tDCS in LLD, which was a safe and effective treatment in alleviating depressive symptoms, with mild cognitive improvements observed. Further larger scale randomized controlled trials are needed to confirm this result.

## 1 INTRODUCTION

### 1.1 Key features of LLD and treatment challenges

Late‐life depression (LLD) is one of the costliest global health issues, with a prevalence rate of up to 16%.^1^ Distinctive from adult‐onset depression, patients with LLD often experience a longer recovery time and receive a less favorable prognosis due to frequent relapses and residual symptoms.^2^ Sleep disturbance, fatigue, and hopelessness about the future are reported more commonly in LLD patients than in their younger counterparts.^3^ Due to the heterogenous clinical profiles and its disabling nature,^4^ the quality of life of those with LLD is greatly compromised.^5^, ^6^ In addition, greater risks of medical comorbidity have been observed in LLD, which in turn, increases one's susceptibility to the side effects of the antidepressants.^7^ Over 50% of people with LLD do not achieve symptomatic remission.^8^ Between 30 and 50% do not respond to one antidepressant trial.^9^ LLD is also closely correlated with cognitive impairments, with executive functioning and memory loss being the most predominant features in LLD.^10^ As the aging population continues to surge, LLD will undeniably create further social and economic burdens on our society.

### 1.2 Limitations of current treatment approaches

Although antidepressants have been well established as the first line of effective treatment for LLD,^11^ up to one‐third of patients with LLD show a suboptimal response or resistance to antidepressant therapy.^12^ Furthermore, their efficacy in addressing depression‐related cognitive deficits remains questionable. Cognitive dysfunctions are consistently coupled with depressive symptoms in LLD.^13^ People with LLD may not return to normal levels of performance, particularly with respect to memory and executive functions, despite remission of depressive symptoms after antidepressant treatments.^14^ Those with memory impairments were more susceptible to developing dementia relative to those without memory deficits.^15^ This implies that antidepressants may not fully address the complex symptomology in LLD. Hence, an alternative treatment approach targeting both depressive and cognitive symptoms is vital in improving the prognosis in LLD. One such approach is transcranial direct current stimulation (tDCS).

### 1.3 Descriptions and possible mechanisms of tDCS

tDCS is a non‐invasive, neurostimulation technique in which a mild direct current (1‐2 mA) is induced through the cerebral cortex via electrodes placed on the scalp, which in turn modifies cortical excitability, depending on the polarity directions.^16^ No severe adverse events have been reported in over 40 previous studies involving the geriatric population.^17^ It is a safe, easily administered, yet affordable, non‐invasive neurostimulation technique, with persistent treatment effects that can last up to an hour.^18^

While the exact mechanisms of tDCS are yet to be understood,^19^ tDCS is said to exert its effects by modulating cortical excitability, which results in alterations in the corresponding cortical functioning and synaptic release probability uptake and sensitivity.^20^ Anodal and cathodal stimulation triggers neuronal depolarization (ie, increased spontaneous firing) and hyperpolarization (ie, decreased neuronal firing), respectively.^21^ Long‐term plasticity is enhanced, with modulations in the rate of neurotransmitter release.^22^

### 1.4 Stimulation Target in Depression—DLPFC

Serotonin deficits and asymmetrical neural activities in the dorsal lateral prefrontal cortex (DLPFC) (ie, hypoactivity and hyperactivity in the left and right DLPFC^23^) are two key neurological abnormalities in depression. tDCS and serotonin are known to enhance one another's functions. tDCS increases the release of serotonin, mediated by serotonin transporters,^24^ while a continuous enhancement of serotonin by antidepressants strengthens the LTP‐like glutamatergic plasticity induced by tDCS.^25^ Moreover, tDCS has been shown to exert its antidepressant effects by modulating the hypoactivity in DLPFC in depression. Brunoni et al^26^ have found a superior effect on treatment response, remission, and reduced depressive symptoms in intervention groups, relative to sham controls, across six randomized controlled trials that administered anodal tDCS at the left DLPFC in depressed adults. Its effect size was comparable to those receiving antidepressants or repetitive transcranial magnetic stimulation.^26^ Similar treatment effects were seen in enhancing working memory^27^ and executive functioning.^28^ Furthermore, a reduction in executive deficits in patients with LLD may indirectly alleviate the depressive symptoms and enhance the treatment response.^29^ Indeed, anodal stimulation over the left prefrontal cortex in schizophrenia patients showed an improvement in the functional capacity and depressive symptoms.^30^ This lends support for tDCS's treatment potential for those with LLD as a monotherapy or augmentation with antidepressants. However, it should be noted that controversial findings observed no antidepressant differences between active and sham tDCS for depression.^31^

### 1.5 HD‐tDCS

As evidence has shown that the highest cortical current density in tDCS might not be induced directly under the target electrode,^32^ the spatial focality of conventional tDCS thus remains questionable. This implies that the treatment efficacy of tDCS could be adversely affected, which might also explain the discrepancy in previous findings.^26^, ^31^

Unlike conventional tDCS, high‐definition tDCS (HD‐tDCS) is typically administered with two or more smaller electrodes. A 4 × 1 ring set‐up would be the most typical design, whereby a central anodal electrode is surrounded by four return cathodal electrodes. The density of the cortical field and spatial focality can be adjusted by altering the diameter of the ring set‐up.^31^ Other strengths of HD‐tDCS over tDCS include longer lasting treatment effects due to a more precise cortical field^25^ and better tolerability.^33^ To our knowledge, no HD‐tDCS study has been performed on patients with LLD.

Although some promising results of tDCS were seen, including a reduction of working memory deficits in LLD,^34^ these results did not include measures for both the depressive and cognitive symptoms, nor was HD‐tDCS administered; only tDCS was administered. Moreover, with little tDCS research on LLD, it is vital to explore the efficacy and tolerability of HD‐tDCS on LLD, a treatment approach that is safe and easy to administer, with proven efficacy in ameliorating depressive symptoms.

### 1.6 Aims

Therefore, we aimed to perform an open‐label pilot study to examine the effectiveness of HD‐tDCS as an augmentation therapy with antidepressants in improving depressive and cognitive symptoms in patients with LLD. Significant improvements were hypothesized in the depressive, cognitive, and daily functioning outcomes across various assessment timepoints.

## 2 METHOD

### 2.1 Study design

This was a 2‐week open‐label study whereby all participants would receive ten sessions of HD‐tDCS (5 consecutive daily sessions of 30 minutes weekly, for a total of 10 sessions) in a psychiatry outpatient clinic in Hong Kong.

### 2.2 Ethical approval

Written informed consent was obtained from all participants. The study was approved by the Institutional Review Board and was conducted in accordance with the Good Clinical Practice and the Declaration of Helsinki. This study was registered on the HKU Clinical Trial Registry (HKUCTR‐2357).

### 2.3 Participants

The participants were recruited between July 2018 and Mar 2019 from a local public psychiatry outpatient clinic. A total of fifteen patients were identified and screened for eligibility by their case medical officers.

Inclusion criteria were as follows: (a) Chinese elderly with an age of 60 or above; (b) a history of major depressive disorder (MDD) (including any major depressive episodes/dysthymia/adjustment disorder/recurrent depressive disorder) meeting the 5th Edition of the Diagnostic and Statistical Manual of Mental Disorders (DSM‐V) criteria^35^; (c) at least mild or above in their severity of depressive symptoms (ie, a total score of ≥7 on HAM‐D‐17); and (d) a stable antidepressant dosage for at least 2 weeks prior to the study, with no changes during the whole study period.

Exclusion criteria were (a) a DSM‐V diagnosis of other than MDD or anxiety disorders (eg, bipolar affective disorder and schizophrenia); (b) a HK‐MoCA score that is below the second percentile according to the subject's age and education level; and (c) any concomitant major medical/neurological conditions or evidence of active infections or significant communicative impairments.

### 2.4 HD‐tDCS procedures

**Figure 1:**
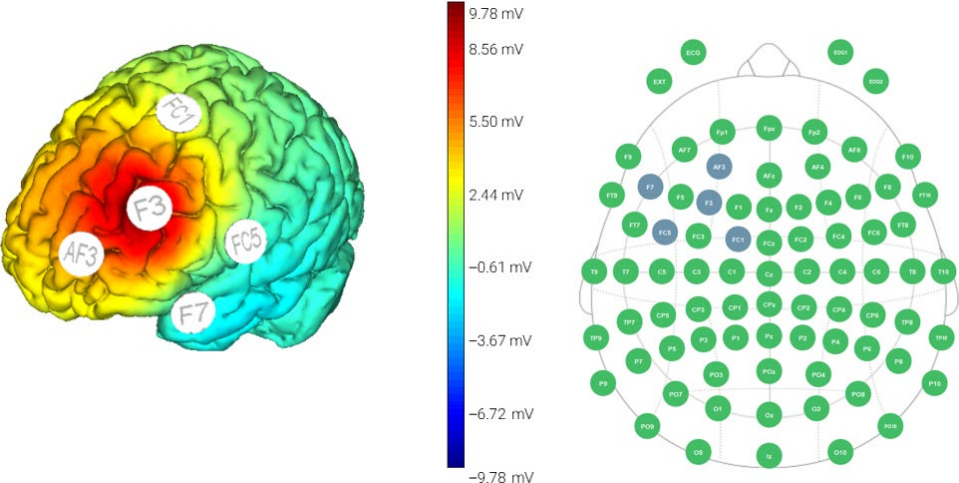
HD‐tDCS Stimulation Preview Map used in present protocol. The anode was positioned over the left DLPFC (F3; 10‐20 EEG system), with the cathode being placed over four positions equidistance from each other and 4.5 cm radius from the anode (namely FC1, AF3, F7 and FC5). A 2 mA stimulation was delivered for 20 min with a gradual ramp up and ramp down of the current over the first 30 s. Each session lasted for around 30 min, with two consecutive weeks of 5‐d treatment sessions weekly

The HD‐tDCS intervention was administered at the psychiatric outpatient clinic. Nursing and supporting staff were available in case of emergencies. The intervention was administered using Starstim® produced by Neuroelectrics. The HD‐tDCS device was controlled wirelessly via the computer, using the Starstim® software. The montages were the 4 × 1 ring set‐up, which is a typical HD‐tDCS stimulation protocol. There was a central anodal electrode surrounded by four return cathodal electrodes. The anode was placed over the left DLPFC, which was located at F3, based on the 10/20 electroencephalogram system. The four cathodal electrodes were placed at FC1, AF3, F7, and FC5, forming a circle with a radius of 4.5 cm (Figure 1). Conductive electrode gel was applied on the scalp at all the designated stimulation areas. To ensure the electrodes were secured in place, a different cap size was used depending on the subject's head size. Prior to each session, impedance checks were performed using the Starstim® software. The participants were instructed to relax for the first 5 minutes of each session during the stimulation set‐up. A 2 mA stimulation was then delivered for 20 minutes, with a gradual increase and decrease of the current over the first 30 seconds. Each patient was asked to relax and do nothing during the intervention. The administrator closely monitored the impedances throughout each session and recorded any side effects experienced by the participants. They were allowed to rest for 5 minutes after the intervention and were systematically asked if they experienced any discomfort. Each session lasted for approximately 30 minutes, and the sessions took place for five consecutive days each week, for two consecutive weeks.

### 2.5 Assessments

All assessments and treatment sessions were administered by a trained research assistant (HLW) or a psychiatrist (PWC). All participants were assessed in terms of their depressive symptoms and cognitive and daily functioning across five timepoints, including the baseline (*t*0), the 5th day of intervention (*t*1) and the 10th day of intervention (*t*2), as well as 1 month (*t*3) and 3 months (*t*4) after the treatment's completion.

### 2.6 Outcome measures

#### 2.6.1 Primary outcomes

Depressive symptoms were assessed by the Hamilton Depressive Rating Scale (HAM‐D17^36^), which is a widely used and reliable measure of depressive symptoms.^37^ The total scores range from 0 to 52, with a higher score suggesting a greater severity in depression. A score of less than seven was defined as remission. A clinical response was defined as a 50% or greater reduction in the HAM‐D total scores from the baseline.

#### 2.6.2 Secondary outcomes

##### Apathy—Clinician‐Rated Apathy Evaluation Scale—Hong Kong version (AES‐C‐HK)

The severity of apathy was assessed using the Hong Kong version of the Clinician‐Rated Apathy Evaluation Scale (AES‐C‐HK) (in press). The AES‐C‐HK is an 18‐item scale that measures apathy as a neuropsychiatric symptom. Its internal consistency was excellent (*α* = .946, Cronbach's alpha). Satisfactory interrater and test‐retest reliability have been reported.

##### Anhedonia—Snaith‐Hamilton Pleasure Scale (SHAPS)

Anhedonia was measured using the Chinese version of the Snaith‐Hamilton Pleasure Scale (SHAPS^38^). SHAPS is a 14‐item, self‐reported questionnaire examining anhedonia for neuropsychiatric disorders, covering four domains of hedonic experience: interest/pastimes, social interaction, sensory experience, and food/drink. The Chinese version has been well validated in previous studies.^38^

##### Cognitive functioning

Global cognitive functioning was assessed by the Cantonese Mini‐Mental State Examination (C‐MMSE^39^) & Montreal Cognitive Assessment Hong Kong version (HK‐MoCA^40^). To minimize practice effects, alternative forms of HK‐MoCA were used across different timepoints. Executive functioning was assessed by the Color‐Word Stroop Test^41^ and Category Verbal Fluency Test.^42^ Attention and the speed of information processing were measured by the Trail Making Test A/B (TMT^43^), and the Forward and Backward Digit Span^44^ was used to measure working memory.

##### Daily functioning

Instrumental activities of daily living (IADL) were examined using the Hong Kong Chinese version of the Lawton Instrumental Activities of Daily Living Scale (IADL‐CV^45^). A total of nine IADL domains were covered, including the ability to use a telephone, go shopping, prepare food, and do housekeeping and laundry tasks, as well as use transportation, manage finances, handle medication, and do handyman work. A higher score indicates greater functioning, meaning that an elderly person can live independently in the community.

##### Adverse effects

A checklist of potential adverse effects associated with the HD‐tDCS administration was generated from available literature reports^46^ (see Appendix S1). To monitor tolerability and any adverse events during the intervention, all participants were asked systematically, at the end of each session, if they had experienced any side effects.

### 2.7 Statistical analysis

Treatment compliance and the descriptive statistics of the demographics and clinical variables at the baseline were reported. All of the statistical tests were two‐tailed, with the significance value set at *P* = .05. Statistical analyses were conducted using the IBM SPSS Statistics for Windows, Version 25.0.^47^

Sets of repeated measures analyses of variance (ANOVA), with one dependent variable per model, were performed to assess the changes in all numerical clinical outcome variables (ie, depressive severity, cognitive, and daily functioning) across all assessment timepoints, with time as an independent variable and with five levels, namely, the baseline (*t*0), week 1 day 5 (*t*1), week 2 day 10 (*t*2), a 1‐month follow‐up (*t*3), and a 3‐month follow‐up (*t*4). A Greenhouse‐Geisser correction was used for corrections in ANOVA if sphericity was violated. If significant main effects of time were found, post hoc pairwise comparisons with Bonferroni corrections were conducted accordingly.

For categorical outcome variables, such as remission rates and the clinical response as indicated by the HAM‐D total scores, two sets of Cochran's *Q* tests were performed to assess the effect of time accordingly. McNemar tests with Bonferroni corrections were also performed as pairwise comparisons between the assessment timepoints, if the effect of time was found to be significant.

## 3 RESULTS

### 3.1 Demographics and treatment compliance

A total of 15 subjects participated in the study, with all subjects having completed all ten of the stimulation sessions. The participants had a mean age of 73.27 (SD = 6.25) years, and 86.67% were female. All of the participants had been on a stable dosage of antidepressants for at least 2 weeks before the study entry. On average, the participants had 16.93 (SD = 15.40) years of depressive symptoms. Further demographic details can be found in Table 1.

**Table 1:** Demographics at study entry (N = 15)

Demographics		n (%)	*M* (SD)
Gender	Female, n (%)	13 (86.67)	
Age	Years old		73.27 (6.25)
Education level	Years of education		4.73 (4.89)
Marital status	Unmarried, n (%)	1 (6.67)	
	Married, n (%)	7 (46.67)	
	Widowed, n (%)	7 (46.67)	
Familial history of mood disorders	Yes, n (%)	4 (26.67)	
Duration of depressive symptoms	Number of years		16.93 (15.40)
Cumulative illness rating scale (CIRS)	Total scores		6.07 (2.37)
	Total cardiovascular risk		1.47 (1.36)

### 3.2 Depressive severity, remission rates, clinical response, levels of apathy, and anhedonia

#### 3.2.1 Depressive severity

A significant effect of time was found in the overall severity of the depressive symptoms, as indicated by the HAM‐D‐17 total scores (*P* < .001; see Table 2). When compared with the severity at the baseline (*M* = 11.83, SD = 5.70), a significantly lower level of severity was seen at *t*1 (*M* = 6.17, SD = 4.45, *P* = .001), *t*2 (*M* = 4.42, SD = 3.18, *P* = .001), *t*3 (*M* = 3.83, SD = 3.59, *P* = .001), and *t*4 (*M* = 5.08, SD = 4.96, *P* = .005).

**Table 2:** Overall changes in depressive symptoms across all timepoints

Domains	Scales	N = 15			N = 14	N = 12	Effects of time				Post‐hoc Bonferroni comparisons
		Baseline (*t*0)	Week 1 Day 5 (*t*1)	Week 2 Day 10 (*t*2)	1 mo FU (*t*3)	3 mo FU (*t*4)					
		*M* (SD)	*M* (SD)	*M* (SD)	*M* (SD)	*M* (SD)	*df*	*F*	*P*	OP	
Depressive symptoms	HAM‐D‐17 total	11.83 (5.70)	6.17 (4.45)	4.42 (3.18)	3.83 (3.59)	5.08 (4.96)	4,44	19.43	<.001***	1	*t*1 < *t*0: *P *= .001*** *t*2 < *t*0: *P *= .001*** *t*3 < *t*0: *P *= .001*** *t*4 < *t*0: *P *= .004**
	HAM‐D‐17 clinical response, n (%)	NA	7 (46.67)	12 (80.00)	12 (85.71)	8 (66.67)	Cochran's *Q*: χ^2^(3) = 5.50, *P *= .139				NA
	HAM‐D‐17 in remission, n (%)	3/15 (20)	11 (73.33)	12 (80.00)	13 (92.90)	10 (83.33)	Cochran's *Q*: χ^2^(4) = 22.889, *P *< .001***				McNemar tests: *t*1 > *t*0: *P *= .008** *t*2 > *t*0: *P *= .004** *t*3 > *t*0: *P *= .002** *t*4 > *t*0: *P *= .016*
	AES‐C‐HK	40.17 (10.21)	34.50 (6.23)	30.50 (6.07)	31.33 (6.87)	28.67 (4.85)	4,44	6.67	0.011*	0.796	*t*2 < *t*0: *P *= .011*
	SHAPS	0.92 (2.27)	0.58 (1.24)	0.17 (0.39)	0.50 (1.00)	0.25 (0.62)		0.907	0.389	0.159	NA

Note

HAM‐D‐17 total = Total Scores in Hamilton Depressive Rating Scale (HAM‐D‐17); HAM‐D‐17 clinical response, n (%) = Percentage of participants with 50% or more reduction in the HAM‐D‐17 total scores from baseline; HAM‐D‐17 in remission, n (%) = Percentage of participants with a total score in HAM‐D‐17 of ≤7; AES‐C‐HK = Total scores in Clinician‐rated Apathy Evaluation Scale Hong Kong version; SHAPS = Total scores in Snaith‐Hamilton Pleasure Scale. * *P*<.05; ** *P*< .01;*** *P* < or = .001

#### 3.2.2 Clinical response

Despite an increasing trend in the percentage of participants with clinical response in terms of their depressive severity (ie, more than a 50% reduction in total HAM‐D scores from the baseline), over time (*t*0 vs *t*1 = 46.67%, *t*0 vs *t*2 = 80.00%, *t*0 vs *t*3 = 85.71%, *t*0 vs *t*4 = 66.67%), the effect of time was not significant (Cochran's *Q*: χ^2^(3) = 5.50, *P* = .139).

#### 3.2.3 Rates of remission

A significant improvement in remission was seen over time (Cochran's *Q*: χ^2^(4)  = 22.89, *P* < .001). McNemar tests with Bonferroni corrections revealed a significantly higher remission rate at *t*1 (73.33%, *P* = .008), *t*2 (80%, *P* = .004), *t*3 (92.90%, *P* = .002) and *t*4 (83.33%, *P* = .011), when compared with that at the baseline (20%).

#### 3.2.4 Apathy and anhedonia

There was a significant effect of time on the levels of apathy (*P* = .011). A significant reduction in apathy scores was observed when comparing them at post (*M* = 30.50, SD = 6.07) and the baseline (*M* = 40.17, SD = 10.21). For anhedonia, as indicated by the total SHAPS scores, no significant effect of time was seen (*P* = .0389).

### 3.3 Neuropsychological & daily functioning

A significant time effect was found in the overall cognitive functioning, as indicated by C‐MMSE (*P* = .016), with a significantly higher functioning score being found at *t*4 (*M* = 26.83, SD = 2.25), relative to that at the baseline (*M* = 25.00, SD = 2.13). For verbal fluency, significant improvements were seen in the 30th (*P* = .025) and 60th total number of items being retrieved in CVFT over time (*P* = .018). More items were recalled in the latter at *t*2 (*M* = 44.42, SD = 7.76), relative to that at the baseline (*M* = 40.08, SD = 6.71, *P* = .015) (see Table 3).

**Table 3:** Overall changes in cognitive and daily functioning across all timepoints

Domains	Scales	N = 15			N = 14	N = 12	Effects of time				Post‐hoc Bonferroni comparisons
		Baseline (*t*0)	Week 1 Day 5 (*t*1)	Week 2 Day 10 (*t*2)	1 mo FU (*t*3)	3 mo FU (*t*4)					
		*M* (SD)	*M* (SD)	*M* (SD)	*M* (SD)	*M* (SD)	*df*	*F*	*P*	OP	
Cognitive functioning	HK‐MoCA	22.42 (3.34)	22.75 (3.96)	23.92 (3.45)	23.25 (4.35)	24.17 (2.79)	4,44	1.13	0.353	0.326	NA
	C‐MMSE	25.00 (2.13)	26.75 (2.22)	26.50 (2.36)	25.92 (2.43)	26.83 (2.25)		3.43	0.015*	0.814	*t*4 > *t*0: *P* = .056 (m.s.)
	Stroop interference	16.88 (6.24)	13.43 (6.70)	14.36 (11.49)	12.67 (6.63)	13.92 (7.05)		0.48	0.748	0.153	NA
	TMT Interference A	65.38 (58.46)	56.70 (44.95)	49.98 (20.79)	54.35 (33.66)	50.33 (29.08)		0.94	0.393	0.174	NA
	TMT Interference B	58.91 (50.34)	54.21 (43.16)	48.75 (21.10)	51.83 (32.63)	50.29 (28.94)		0.446	0.775	0.175	NA
	Forward DS Span Length	7.92 (1.31)	7.92 (0.79)	7.75 (1.29)	8.00 (1.04)	7.67 (1.37)		0.376	0.824	0.128	NA
	Forward DS Total	10.42 (2.35)	10.58 (2.23)	10.50 (2.20)	10.83 (1.95)	9.83 (2.98)		1.011	0.412	0.293	NA
	Backward DS Span Length	4.17 (1.70)	4.33 (2.10)	4.42 (2.31)	4.67 (1.87)	4.33 (1.87)		0.611	0.556	0.140	NA
	Backward DS Total	5.25 (3.08)	5.92 (3.78)	5.83 (4.28)	6.33 (3.14)	5.92 (3.58)		0.865	0.492	0.253	NA
	CVFT ‐ 30th Total	27.42 (5.23)	29.67 (5.19)	30.33 (5.21)	31.42 (7.65)	31.42 (6.40)		3.092	0.025*	0.767	No significant pairwise comparisons
	CVFT ‐ 60th Total	40.08 (6.71)	41.50 (6.84)	44.42 (7.76)	45.17 (10.18)	43.75 (8.50)		3.321	0.018*	0.800	*t*0 vs *t*2: *P *= .015*
Daily functioning	Chinese IADL	26.33 (1.37)	26.58 (0.79)	25.17 (3.79)	25.67 (2.87)	25.75 (3.17)	4,44	1.425	0.261	0.212	NA

Note

*HK‐MoCA* = Total score in Montreal Cognitive Assessment Hong Kong version; *C‐MMSE* = Total score in Cantonese Mini‐Mental State Examination; *Stroop Interference* = Reaction time (in seconds) in Stroop III—[(Reaction time in Stroop I + Reaction time in Stroop II)/2]; *TMT Interference A* = Interference in Trail Making Test: difference in Reaction Time (in seconds) between test using Arabic numbers and the Test using alternating Arabic and Chinese Numbers; *TMT Interference B* = Interference in Trail Making Test: difference in Reaction Time (in seconds) between test using Chinese numbers and the Test using alternating Arabic and Chinese Numbers*; Forward DS Span Length* = Total Span Length in Forward Digit Span Test; *Forward DS Total* = Total scores in Forward Digit Span Test; *Backward DS Span Length* = Total Span Length in Backward Digit Span Test; *Backward DS Total* = Total scores in Backward Digit Span Test; *CVFT ‐ 30th Total = *Total number of items being recalled at the 30‐s interval at the Category Verbal Fluency Test; *CVFT—60th Total* = Total number of items being recalled at the 30‐s interval at the Category Verbal Fluency Test*; Chinese IADL = *Total scores in Chinese Lawson Instrumental Activities of Daily Living. *P<.05

No time effects were found in overall cognitive functioning, as indicated by HK‐MoCA total scores (*P* = .353).

For executive functioning and attention, the effects of time were not significant for the following measures: Stroop interference score, *P* = .748, TMT interference score (Alternate RT—Roman Number RT), *P* = .393, and TMT interference score (Alternate RT—Chinese Number RT), *P* = .775. Similarly, none of the time effects were significant, as seen in the performance on working memory (*P *> .05). For daily functioning as indicated by the Chinese Lawton IADL total scores, no significant effect of time was seen (*P *> .05).

### 3.4 Adverse outcomes and side effects

Nine subjects reported mild side effects, such as tingling, itchiness, and mild skin redness at the stimulation site, with no adverse outcomes being reported. Treatment compliance was excellent, with no dropouts being seen in the 2‐week stimulation phase, suggesting good tolerability of the intervention.

## 4 DISCUSSION

Our open‐label pilot study has demonstrated the treatment efficacy of 2 weeks of HD‐tDCS stimulation at DLPFC (five consecutive days of 30‐minute sessions for 2 weeks) as an augmentation therapy with antidepressants in ameliorating the depressive symptoms and severity, along with mild enhancements in overall cognitive functioning and verbal fluency. Excellent tolerability of HD‐tDCS was also indicated, as no serious adverse effects were reported.

In particular, the improvements in the overall depressive severity and the remission rates were not only seen at the pre‐post phase, but also were maintained at the 1‐month and 3‐month follow‐up, implying sustained treatment effects. Although the proportion of participants with a clinical response did not significantly increase over time, a growing trend was found. The levels of apathy, but not anhedonia, were alleviated within the pre‐post treatment phase.

A neural explanation for the reduction in depressive symptoms is the modulation of the asymmetrical activation in the DLPFC by HD‐tDCS (ie, repetitive sessions of anodal stimulation normalizing the hypoactivity in the left DLPFC). This is well supported by previous literature,^26^ which has shown the superior effectiveness of active tDCS in improving treatment response, remission, and depressive symptoms in the intervention group, relative to sham controls, across six randomized controlled trials that administered anodal tDCS at the left DLPFC in depressed adult patients. Further support could be lent from Brunoni et al,^48^ who found that a 6‐week combined treatment of tDCS and sertraline produced a quicker treatment response relative to the those who received either tDCS or sertraline solely. Although our study was an open‐label study without controls, the direction of our findings aligns with that in the previous literature, suggesting the potential additive therapeutic benefits of tDCS intervention when used in conjunction with antidepressants.

Although mild cognitive enhancements were seen in overall cognitive functioning and verbal fluency, contrary to our hypotheses, no improvements were seen in executive functioning over time as a result of the 2‐week HD‐tDCS intervention. Similarly, some studies have also found that active tDCS produced no cognitive enhancement in depression.^49^, ^50^ This might be explained in terms of the relatively high functioning cognitive profiles in our current sample, as indicated in the mean C‐MMSE and HK‐MoCA total scores in Table 2, in which any subtle enhancements in executive functioning might not be captured within a short period, due to the ceiling effect. Previous research has also suggested that psychiatric patients with greater cognitive deficits (eg, those with schizophrenia) would exhibit more pronounced cognitive improvements in working memory upon receiving tDCS treatment.^51^ As our present sample involved LLD patients with a relatively mild depressive profile, it is possible that any subtle cognitive enhancements might not be observed.

### 4.1 Strengths of the present study

To the best of our knowledge, the present study is the first to examine both the short‐ and long‐term therapeutic efficacy of HD‐tDCS as an augmentation therapy with antidepressants in ameliorating both depressive and cognitive symptoms in patients with LLD.

Furthermore, we have attempted to operationalize the primary outcome of our present study—that is, defining depressive symptoms by three levels, namely, the overall severity, the clinical response, and the rates of remission. This is to allow comparisons to be made with previous literature regarding the treatment efficacy of tDCS on reducing depressive symptoms. As research has suggested, the conflicting findings could be partially explained in terms of the diverse definitions used in operationalizing the levels of depressive symptoms.^52^ For instance, tDCS was only found to be effective in improving depressive symptoms than sham controls when standard depression scales were used,^53^ but not when remission rates or clinical responses were adopted as outcome measures.^54^

It is also hoped that our initial findings will provide some insight and framework for designing future studies on HD‐tDCS to reduce both depressive and cognitive symptoms in patients with LLD, whether it be the stimulation protocol (ie, the stimulation sites, the frequency or duration of the sessions, or the current strengths) or the types of outcome measures being used.

### 4.2 Limitations of the current study

Despite the promising results, our study had several methodological limitations. Due to the use of a small sample size and an open‐label pilot study design, the interpretation of our present findings is limited due to its exploratory nature. Despite alternative versions of cognitive tests being used wherever possible (eg, HK‐MoCA), practice effects might come into play when examining the improvements in verbal fluency and overall cognitive ability, as seen in our current study. A stable antidepressant dosage for at least 2 weeks prior to the study may not totally exclude the possibility of the effect from the change of medications, which may result in an overestimation of the effect by tDCS. Moreover, the diversity in the clinical profiles might act as potential confounds in influencing the validity of our findings, whether it be the types of antidepressants being used, the degree of treatment resistance, or the duration of the depressive symptoms. Yet, any subgroup analyses might not be possible, due to the small sample size being used in the present study. The use of multiple domains as to represent cognitive functioning may have resulted in type I error. Nevertheless, the advantage of using multiple domains is that it delineates different domains of cognitive outcomes. In addition, the adoption of an open‐label design means our present findings might be susceptible to a placebo effect.^55^

### 4.3 Implications for future research

Thus, to counteract the aforementioned limitations, future research should adopt a randomized controlled trial design with a larger sample size, with control arms that involve treatment‐as‐usual (or on antidepressants only) or other effective interventions that target cognitive dysfunctions in LLD, such as cognitive training,^56^ in order to determine if HD‐tDCS alone or a combined treatment will maximize the therapeutic benefits for patients with LLD.

## 5 CONCLUSION

In conclusion, the current study has shown that HD‐tDCS was effective in eliciting improvements in the depressive symptoms with mild cognitive enhancements. Future studies should aim for a larger scale, randomized controlled trial in determining the optimal stimulation protocol and the clinical profiles, which could best benefit from HD‐tDCS in reducing the hallmarks of LLD—the depressive symptoms and cognitive dysfunctions that play a contributing role in the prognosis and the quality of life in these patients.

## CONFLICT OF INTEREST

The authors declare no conflict of interest.
