# Supplementary material for: High‐definition transcranial direct current stimulation—An open‐label pilot intervention in alleviating depressive symptoms and cognitive deficits in late‐life depression
Source: CNS Neurosci Ther. 2019 Oct 28;25(11):1244–53. doi: 10.1111/cns.13253 (PMC6834921; doi:10.1111/cns.13253)
Supplement: Supplementary file 1 [file CNS-25-1244-s001.pdf]

HDtdcs Adverse Effects Checklist

**Title of project**	**HD-tDCS as augmentation therapy in late-life depression (LLD) – A pilot study**										
**Participant No**											
	**Week 1**										
**After session**	**1**		**2**		**3**		**4**		**5**		**After session**
**Date**	**/ /2018**		**/ /2018**		**/ /2018**		**/ /2018**		**/ /2018**		**Date**
	**Severity**	**Related to HDtdcs?**	**Severity**	**Related to HDtdcs?**	**Severity**	**Related to HDtdcs?**	**Severity**	**Related to HDtdcs?**	**Severity**	**Related to HDtdcs?**	
Tingling*											Tingling*
Itching*											Itching*
Skin redness*											Skin redness*
Burning sensation*											Burning sensation*
Headache*											Headache*
Neck pain*											Neck pain*
Scalp pain*											Scalp pain*
Numbness											Numbness
Fatigue after stimulation/ Sleepiness*											Fatigue after stimulation/ Sleepiness*
Dizziness											Dizziness
Nausea											Nausea
Trouble concentrating*											Trouble concentrating*
Acute mood change*											Acute mood change*
A phosphene (i.e. a brief flash of light) at the start of stimulation											A phosphene (i.e. a brief flash of light) at the start of stimulation
Others (Please specify)											Others (Please specify)
Remarks											Remarks

Severity?	1	absent	2	mild	3	moderate	4	severe		
AE related to HDtdcs?	1	none	2	remote	3	possible	4	probable	5	definite

*also in Brunoni et al 2011 Tdcs AE qnr

HDtdcs Adverse Effects Checklist											
**Title of project**	**HD-tDCS as augmentation therapy in late-life depression (LLD) – A pilot study**										
**Participant No**											
	**Week 2**										
**After session**	**1**		**2**		**3**		**4**		**5**		**After session**
**Date**	**/ /2018**		**/ /2018**		**/ /2018**		**/ /2018**		**/ /2018**		**Date**
	**Severity**	**Related to HDtdcs?**	**Severity**	**Related to HDtdcs?**	**Severity**	**Related to HDtdcs?**	**Severity**	**Related to HDtdcs?**	**Severity**	**Related to HDtdcs?**	
Tingling*											Tingling*
Itching*											Itching*
Skin redness*											Skin redness*
Burning sensation*											Burning sensation*
Headache*											Headache*
Neck pain*											Neck pain*
Scalp pain*											Scalp pain*
Numbness											Numbness
Fatigue after stimulation/ Sleepiness*											Fatigue after stimulation/ Sleepiness*
Dizziness											Dizziness
Nausea											Nausea
Trouble concentrating*											Trouble concentrating*
Acute mood change*											Acute mood change*
A phosphene (i.e. a brief flash of light) at the start of stimulation											A phosphene (i.e. a brief flash of light) at the start of stimulation
Others (Please specify)											Others (Please specify)
Remarks											Remarks

Severity?	1	absent	2	mild	3	moderate	4	severe		
AE related to HDtdcs?	1	none	2	remote	3	possible	4	probable	5	definite

*also in Brunoni et al 2011 Tdcs AE qnr
